# Prenatal Arsenic Exposure and DNA Methylation in Maternal and Umbilical Cord Blood Leukocytes

**DOI:** 10.1289/ehp.1104173

**Published:** 2012-03-30

**Authors:** Molly L. Kile, Andrea Baccarelli, Elaine Hoffman, Letizia Tarantini, Quazi Quamruzzaman, Mahmuder Rahman, Golam Mahiuddin, Golam Mostofa, Yu-Mei Hsueh, Robert O. Wright, David C. Christiani

**Affiliations:** 1 Oregon State University, College of Public Health and Human Sciences, Corvallis, Oregon, USA; 2 Harvard School of Public Health, Boston, Massachusetts, USA; 3 Center of Molecular and Genetic Epidemiology, Department of Environmental and Occupational Health, Ca’ Granda Ospedale Maggiore Policlinico IRCCS Foundation, University of Milan, Milan, Italy; 4 Dhaka Community Hospital, Dhaka, Bangladesh; 5 Department of Public Health, School of Medicine, College of Medicine, Taipei Medical University, Taipei, Taiwan

**Keywords:** Alu, arsenic, developmental programming, DNA methylation, environmental exposures, epigenetics, *in utero* exposure, LINE-1, p16, p53

## Abstract

Background: Arsenic is an epigenetic toxicant and could influence fetal developmental programming.

Objectives: We evaluated the association between arsenic exposure and DNA methylation in maternal and umbilical cord leukocytes.

Methods: Drinking-water and urine samples were collected when women were at ≤ 28 weeks gestation; the samples were analyzed for arsenic using inductively coupled plasma mass spectrometry. DNA methylation at CpG sites in *p16* (*n* = 7) and *p53* (*n* = 4), and in LINE-1 and Alu repetitive elements (3 CpG sites in each), was quantified using pyrosequencing in 113 pairs of maternal and umbilical blood samples. We used general linear models to evaluate the relationship between DNA methylation and tertiles of arsenic exposure.

Results: Mean (± SD) drinking-water arsenic concentration was 14.8 ± 36.2 μg/L (range: < 1–230 μg/L). Methylation in LINE-1 increased by 1.36% [95% confidence interval (CI): 0.52, 2.21%] and 1.08% (95% CI: 0.07, 2.10%) in umbilical cord and maternal leukocytes, respectively, in association with the highest versus lowest tertile of total urinary arsenic per gram creatinine. Arsenic exposure was also associated with higher methylation of some of the tested CpG sites in the promoter region of *p16* in umbilical cord and maternal leukocytes. No associations were observed for Alu or *p53* methylation.

Conclusions: Exposure to higher levels of arsenic was positively associated with DNA methylation in LINE-1 repeated elements, and to a lesser degree at CpG sites within the promoter region of the tumor suppressor gene *p16*. Associations were observed in both maternal and fetal leukocytes. Future research is needed to confirm these results and determine if these small increases in methylation are associated with any health effects.

Inorganic arsenic (As) is ubiquitous in the environment, and individuals can be exposed to As from mining and smelting metal ores, pesticide manufacturing and application, and wood preservatives (Mandal and Suzuki 2002). For the general public, ingestion of As-contaminated food and drinking water is the primary route of exposure (Mandal and Suzuki 2002). Currently, populations in Southeast Asia are among the most likely to be exposed to As due to the use of contaminated groundwater for drinking water, with tens of millions of people exposed to As in Bangladesh (Alam et al. 2002). Other countries including Mexico, Chile, Argentina, and the United States also have regions using groundwater for consumption that is contaminated with naturally occurring As (Amini et al. 2008).

Chronic exposure to As is associated with increased risk of cancer and neurological, cardiovascular, respiratory, hepatic, and hematological disease (Vahter 2008). Epidemiological studies show that chronic exposure to As is associated with an increased risk of mortality from cardiovascular disease, infectious disease, and cancer (Sohel et al. 2009). Inorganic As is classified as a known human carcinogen (Bates et al. 1992) but it is not a potent mutagen. When As is administered alone it does not produce tumors in traditional animal models, but it can act as a carcinogen in animal models using fetal exposure paradigms because As crosses the placenta (National Research Council 2001; Tokar et al. 2011b). Transplacental studies in mice show that the offspring of dams who were given 0, 42.5, and 85 ppm As via drinking water from gestational day 8 to 18 (last two-thirds of pregnancy) had a dose-dependent increase in liver, lung, ovary, and adrenal tumors when they reached adulthood (Waalkes et al. 2003, 2004). Furthermore, mice that received As exposure *in utero* and throughout their life course developed more frequent and aggressive tumors at much lower doses compared with mice who only received As exposure during the gestational period (Tokar et al. 2011a).

These studies generated considerable interest in the potential for As to alter epigenetic programming in the fetus (Barker 1992; Edwards and Myers 2007; Jirtle and Skinner 2007; Waterland and Michels 2007; Wu et al. 2004). Because DNA methylation patterns are established during embryogenesis and play an important role in gene transcription, chromosomal stability, X-chromosome inactivation, tissue differentiation, and suppression of repetitive DNA sequences, permanently altering fetal DNA methylation is a potential mechanism linking *in utero* exposures to chronic diseases in adulthood (Geiman and Muegge 2010; Sasaki and Matsui 2008). Moreover, animal models show that DNA methylation in fetal tissues can be altered by arsenic, maternal diet, bisphenol A, vinclozolin, and ethanol, and that the changes in DNA methylation are associated with a shift in the distribution of adult phenotypes (Dolinoy et al. 2006, 2007; Kaminen-Ahola et al. 2010; Waterland and Jirtle 2003; Xie et al. 2007).

Epidemiological studies in adults have observed that chronic arsenic exposure from drinking contaminated water is associated with increased methylation in DNA extracted from whole blood leukocytes (Chanda et al. 2006; Majumdar et al. 2010; Pilsner et al. 2007; Smeester et al. 2011). Yet little is known about how *in utero* exposures to As affects DNA methylation, or how As exposure affects methylation in healthy individuals. Therefore, we examined the association between drinking-water As exposure and DNA methylation in paired maternal and umbilical cord leukocytes using data collected from a prospective birth cohort in Bangladesh. The outcome of this analysis was the percentage of methylated cytosines (%mC) in the promoter regions of two tumor suppressor genes (*p16* and *p53*) that act as checkpoints in the cell cycle, and in two repetitive elements [LINE-1 (long interspersed nucleotide elements) and Alu (short interspersed elements)]. These repeated elements account for approximately 25% of the human genome and are heavily methylated, which is presumed to silence their transcription (Kochanek et al. 1993; Rangwala et al. 2009).

## Methods

*Subject selection and recruitment.* In this analysis we used samples collected from a prospective birth cohort recruited in Sirajdikhan Upazila of Bangladesh. The objective of this cohort was to observe the effects of chronic low-level As exposure on reproductive outcomes. Groundwater testing by the British Geological Survey indicated that this area was moderately As contaminated (British Geological Survey 2001). Additionally, Dhaka Community Hospital (DCH) directs arsenic awareness programs in this area that provide tubewell testing and encourage people to drink only from tubewells that comply with the Bangladesh drinking-water standard of < 50 μg As/L.

DCH-trained health care workers who live in the villages serviced by the clinic identified pregnant women and invited them to join the study. Women were eligible to participate if they were ≥ 18 years of age, had an ultrasound-confirmed singleton pregnancy of < 28 weeks gestation, used a tubewell that supplied groundwater as their primary drinking-water source, planned to live at their current residence for the duration of the pregnancy and continue prenatal health care with DCH, and agreed to deliver at DCH or at home with a DCH-trained midwife. An additional criterion for this analysis was that the participant must have used the same tubewell for ≥ 6 months before enrollment. Study staff administered questionnaires that collected sociodemographic information, medical histories, and other covariates at the time of enrollment. As an incentive, all participants were provided with free prenatal care from DCH and prenatal vitamins that were replenished during monthly checkups in the participant’s home. Informed consent was provided by all participants before enrollment. This study was approved by the Human Research Committees at the Harvard School of Public Health, DCH, and Oregon State University.

*Water As.* At the time of enrollment, a water sample was collected from the tubewell that each participant identified as their primary source of drinking water. Briefly, water samples were collected in 50-mL polypropylene tubes (BD Falcon; BD Bioscience, Bedford, MA, USA) and preserved with reagent grade HNO_3_ (Merck, Germany) to a pH < 2. Samples were kept at room temperature before analysis by inductively coupled plasma–mass spectrometry following U.S. Environmental Protection Agency method 200.8 (Environmental Laboratory Services, North Syracuse, NY, USA). The average (± SD) percent recovery of As from PlasmaCAL multi-element QC standard #1 solution (SCP Science, Canada) was 102% ± 7%. Of the 114 samples included in this analysis, 51 (44.7%) had an As concentration below the 1-μg As/L limit of detection (LOD).

*Urinary As.* At the time of enrollment, participants provided a spot urine sample during their clinical visit. Briefly, urine was frozen at –20°C and shipped on dry ice to Taipei Medical University for analysis following protocols described by Hsueh et al. (1998). Arsenite (As^III^), arsenate (As^V^), monomethylarsonic acid (MMA), and dimethylarsinic acid (DMA) were quantified by high-performance liquid chromatography (Waters 501; Waters Associates, Milford, MA, USA) and hydride-generated atomic absorption spectrometry (Flow Injection Analysis System 400AA 100; Perkin-Elmer, Waltham, MA, USA). Total urinary arsenic (TUA) was calculated by adding As^III^ + As^V^ + MMA + DMA. This approach eliminates interference from arsenobetanine and arsenocholine, which are organic As species found in fish and shellfish.

The average LOD was 0.05 μg/L. The average (± SD) percent recovery of spiked samples was 98.9% ± 6.5%, 100% ± 6.5%, 99.9% ± 6.4%, and 100.1% ± 6.5% for As^III^, As^V^, MMA, and DMA, respectively. The average percent difference of replicate samples composed of standard As solutions was –1.0% ± 3.5%, 0% ± 3.9%, –0.3% ± 3.4%, and –1.3% ± 3.4% for As^III^, As^V^, MMA, and DMA. Urinary creatinine was measured using the kinetic Jaffe method with a Hitachi 7170S autoanalyzer (Hitachi, Tokyo, Japan). Creatinine-adjusted urinary arsenic concentrations were derived by dividing total urinary arsenic (micrograms per liter) by creatinine (grams per liter).

*DNA methylation.* Whole blood was collected in an EDTA-coated vacutainer (BD Scientific, Franklin Lakes, NJ, USA) from the mother at the time of enrollment and at 28 weeks gestation via venipucture. Blood was collected from the umbilical cord shortly after birth via a syringe or by milking the cord. DNA was extracted from 4 mL whole blood using Puregene DNA isolation solutions following manufacturer’s instructions (Qiagen/Gentra Systems, Minneapolis, MN, USA). DNA was stored at 4°C.

DNA methylation analyses were performed on bisulfite-treated DNA using a quantitative assay based on PCR (polymerase chain reaction)–pyrosequencing. The detailed method, location of the gene promoters, amplified regions, and CpG sites that were evaluated have been published previously (Kile et al. 2010). Briefly, DNA was treated using the EZ-96 DNA Methylation-Gold™ Kit (Zymo Research, Orange, CA, USA). Then DNA was amplified using bisulfite-PCR. The PCR product underwent pyrosequencing using the PyroMark™ Q96 MD Pyrosequencing System (Pyrosequencing, Inc., Westborough, MA) using sequencing primers described by Pavanello et al. (2009) and are provided in Supplemental Material, Tables 1 and 2 (http://dx.doi.org/10.1289/ehp.1104173). The percentage of methylated and unmethylated cytosines was quantified for 3 CpG sites in Alu, three CpG sites in LINE-1, seven CpG sites in *p16,* and four CpG sites in *p53.* Methylation was expressed as the %mC over the sum of methylated and unmethylated cytosines in the CpG sites tested. Each marker was pyrosequenced in two replicates and the results averaged.

**Table 1:** General description of the population providing maternal and umbilical cord blood (n = 113).

Variable	n	Mean ± SD	Minimum	Maximum
Cesarean section (yes)		6 (5.3%)						
Premature (< 37 weeks)		13 (11.5%)						
Infant sex (male)		50 (44.3%)						
Prenatal vitamins (no)		2 (1.8%)						
Maternal age (years)		113		23.5 ± 4.2		18		38
Maternal BMI		113		21.0 ± 3.1		13.4		29.0
Birth weight (g)		113		2,786 ± 335		1,740		3,520
Gestational age at enrollment (weeks)		113		13.3 ± 4.8		4.7		25.9
Maternal drinking-water As (μg/L)		113		14.8 ± 36.2		< 1		230
Total urinary As (μg/L)		113		25.3 ± 260.3		0.05		260.3
Total urinary As (μg/g creatinine)		113		1.01 ± 2.6		0.004		21.87
Urinary creatinine (mg/dL)		113		35.6 ± 31.7		3.4		172.8
Cord blood leukocytes (%mC)								
LINE-1		109		80.6 ± 1.9		76.2		85.3
Alu		113		25.0 ± 0.8		76.0		85.1
p16 position 1		112		2.4 ± 1.4		0.87		9.17
p16 position 2		112		2.8 ± 1.3		1.08		6.98
p16 position 3		112		1.4 ± 0.8		0		6.09
p16 position 4		112		2.1 ± 1.0		0.85		5.82
p16 position 5		112		2.1 ± 0.9		1.06		7.66
p16 position 6		112		1.3 ± 0.8		0		5.03
p16 position 7		112		2.4 ± 1.1		0.88		6.60
p53 position 1		103		2.5 ± 0.9		0		7.91
p53 position 2		103		7.5 ± 2.8		2.95		24.13
p53 position 3		103		2.5 ± 1.3		0		13.22
p53 position 4		103		3.7 ± 1.5		0		12.58
Maternal blood leukocytes (%mC)								
LINE-1		101		80.2 ± 2.1		76.0		85.1
Alu		103		25.2 ± 0.7		25.5		27.5
p16 position 1		101		2.6 ± 1.5		0.60		10.87
p16 position 2		101		3.1 ± 1.7		1.05		12.51
p16 position 3		101		1.4 ± 0.6		0		3.21
p16 position 4		101		2.2 ± 1.0		0.73		5.45
p16 position 5		101		2.2 ± 0.7		1.23		4.54
p16 position 6		101		1.2 ± 0.7		0		3.22
p16 position 7		101		2.9 ± 2.2		1.06		16.73
p53 position 1		91		2.8 ± 1.7		1.40		15.59
p53 position 2		91		8.3 ± 2.7		3.91		18.84
p53 position 3		91		2.8 ± 1.0		1.57		7.03
p53 position 4		91		3.8 ± 1.5		0		8.6

**Table 2:** Distribution of water and urinary arsenic concentrations.

Percentile												
Minimum	10th	25th	50th	75th	90th	Maximum
Drinking Water As (μg/L)		< 1		< 1		< 1		1.2		3.7		61		230
Total urinary As (μg/L)		0.05		2.67		5.95		12.35		29.54		60.30		260.29
Total urinary As (μg As/g creatinine)		0.004		0.18		0.3		0.48		0.80		1.42		21.87


Non-CpG cytosine residues were used to verify bisulfite conversion. The success of pyrosequencing in the umbilical cord samples was 100%, 97%, 99%, and 91% for Alu, LINE-1, *p16*, and *p53*, respectively. The success of pyrosequencing in the maternal samples from blood drawn at the time of enrollment was 76%, 76%, 75%, and 61% for Alu, LINE-1, *p16*, and *p53*. To increase the number of maternal samples, DNA methylation results for blood collected at 28 weeks gestational age was substituted for the failed samples. Subsequently, maternal samples include blood drawn at the time of enrollment and at 28 weeks gestational age.

*Statistical analysis.* Drinking-water As concentrations and total urinary arsenic adjusted for creatinine were categorized into tertiles. The %mC was averaged across the three CpG sites evaluated in LINE-1 and in Alu, and the average values were used to represent LINE-1 and Alu methylation (respectively) in all statistical analysis. In contrast, the seven CpG sites evaluated in *p16* and the four CpG sites in *p53* were each evaluated separately. It is unknown whether all CpG sites within promoter regions are equally susceptible to environmental exposures such as As. Therefore, we chose to evaluate the association between arsenic and individual CpG site in the promoter regions of *p16* and *p53* instead of using an average methylation within each promoter region. Improving our understanding of the sensitivity of CpG sites in promoter regions has implications for some commercially available methylation arrays that select only one or more CpG sites within the promoter region of genes.

We calculated descriptive statistics for all DNA methylation outcomes, As exposures, and selected subject characteristics. Spearman correlation coefficients were used to evaluate the relationship between water As and creatinine-adjusted total urinary arsenic (TUA/g). General linear regression models were used to estimate the association between DNA methylation (%mC) at each site and As exposure via drinking-water As (micrograms per liter) or TUA (micrograms per gram creatinine), with each exposure categorized by tertiles. Additional covariates were evaluated in the models including body mass index (BMI; continuous), maternal age (years), infant sex, delivery method (vaginal/cesarean), gestational age (weeks), and prematurity (< 37 weeks/≥ 37 weeks). Only maternal BMI and prematurity were significantly associated with methylation and were included in the final adjusted models. Additionally, we evaluated the association between total urinary arsenic (micrograms per liter) and DNA methylation in models that included creatinine as an independent variable [see Supplemental Material, Table 3 (http://dx.doi.org/10.1289/ehp.1104173)]. All analyses were performed in SAS version 9.1 (SAS Institute Inc., Cary, NC, USA).

**Table 3:** Estimates and 95% confidence intervals (CIs) from general linear regression models that compared DNA methylation (%mC) in leukocytes according to tertiles of drinking-water As (μg/L) and TUA/g (μg/g).

Drinking-water As									TUA/g								
Crude				Adjusted				Crude				Adjusted			
β (95% CI)		p-Value	β (95% CI)		p-Value	β (95% CI)		p-Value	β (95% CI)		p-Value
Cord blood																						
LINE-1		High		0.79	(–0.01, 1.60)		0.05		0.75	(–0.02, 1.53)		0.06		1.56	(0.70, 2.41)		0.001		1.36	(0.52, 2.21)		0.002
		Med		1.15	(0.19, 2.11)		0.02		1.10	(0.17, 2.04)		0.02		0.37	(–0.46, 1.20)		0.38		0.33	(–0.48, 1.14)		0.43
		Low		Ref					Ref					Ref					Ref			
Alu		High		–0.26	(–0.57, 0.06)		0.11		–0.25	(–0.57, 0.07)		0.13		–0.01	(–0.35, 0.34)		0.97		–0.02	(–0.38, 0.34)		0.91
		Med		–0.19	(–0.57, 0.19)		0.33		–0.16	(–0.55, 0.23)		0.43		–0.15	(–0.50, 0.20)		0.39		–0.17	(–0.51, 0.18)		0.35
		Low		Ref					Ref					Ref					Ref			
Maternal blood																						
LINE-1		High		0.66	(–0.27, 1.59)		0.16		0.63	(–0.29, 1.55)		0.18		1.13	(0.11, 2.15)		0.03		1.08	(0.07, 2.10)		0.04
		Med		0.62	(–0.54, 1.78)		0.29		0.53	(–0.62, 1.69)		0.36		0.12	(–0.88, 1.12)		0.81		0.13	(–0.87, 1.12)		0.80
		Low		Ref					Ref					Ref					Ref			
Alu		High		–0.19	(–0.51, 0.13)		0.24		–0.21	(–0.53, 0.11)		0.20		–0.07	(–0.42, 0.29)		0.71		–0.09	(–0.44, 0.27)		0.63
		Med		–0.16	(–0.56, 0.25)		0.44		–0.18	(–0.59, 0.22)		0.38		0.27	(–0.08, 0.62)		0.12		0.28	(–0.07, 0.62)		0.12
		Low		Ref					Ref					Ref					Ref			
Abbreviations: Med, medium; Ref, reference. Models are adjusted for maternal BMI (continuous). Additionally, cord blood models are adjusted for prematurity (< 37 or ≥ 37 weeks gestation).																						

## Results

In this analysis we used data from 114 paired maternal and umbilical cord blood samples. These samples were selected because the mothers and their infants were healthy, there were complete covariate data, and DNA was available from both mother and infant. Of these participants, one individual had a drinking-water As level that was much higher (734 μg As/L) than the next highest value (230 μg As/L). This sample was an outlier, and consequently this mother and her infant were excluded from the analysis, resulting in a final sample size of 113 mother–infant pairs.

All participants used groundwater as their primary source of drinking water for at least 6 months before enrollment. A general description of the population and DNA methylation outcomes are presented in Table 1. Ultrasounds were used to confirm pregnancy and estimate gestational age, which was, on average (± SD), 13.3 ± 4.8 weeks (range, 4.8–25 weeks) at the time of enrollment. On average, mothers were 23.5 years old with a BMI of 21.0 (range, 13.4–29.0), although 20.2% of the mothers were underweight (BMI < 18.5). The average birth weight was 2,786 g (range, 1,740–3,520 g), and 11.5% of infants were premature (< 37 weeks). The vast majority of the births were vaginal and took place at home in the presence of a midwife (94.5%), with the remainder delivered by cesarean section in the hospital. There were also fewer male infants than expected (44.3%). All women were given prenatal vitamins and encouraged to take them daily, but two women reported that they did not take the vitamins because of nausea or a preference not to take medication.

Overall, arsenic exposures were modestly elevated in this population (Table 2), which is consistent with expectations for residents of villages participating in arsenic remediation programs. At enrollment, drinking-water As concentrations ranged from < 1 to 230 μg/L with a median concentration of 1.2 μg/L. Maternal total urinary arsenic concentrations adjusted for creatinine (TUA/g) were strongly correlated with maternal drinking-water As (ρ = 0.32, *p* = 0.0006). Arsenic exposures were categorized into tertiles for statistical analysis. For drinking-water As, the low (< 1 μg/L), medium (1–1.9 μg/L), and high tertiles (1.9–230 μg/L) had average As of 0.5, 1.4, and 42.3 μg/L, respectively. For TUA/g, the low (< 0.35 μg/g), medium (0.35–0.65 μg/g), and high (0.66–21.87 μg/g) tertiles had average As of 0.22, 0.50, and 2.35 μg/g, respectively. The distribution of As in this population resulted in a very small concentration difference between the low and medium tertiles of As exposure.

**Figure 1:**
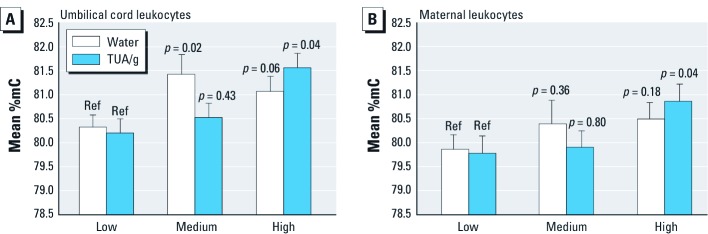
The average %mC in LINE-1 repeated elements was estimated from general linear regression models for each tertile of As exposure measured in drinking-water As and TUA/g for (*A*) umbilical cord leukocytes adjusted for average maternal BMI (21.0) and prematurity (≥ 37 weeks gestation) and (*B*) maternal leukocytes adjusted for average maternal BMI (21.0). *p*-Values evaluate the difference in LINE-1 methylation levels at each tertile of As exposure compared to the lowest tertile (Ref, reference). Error bars represent SEs.

*LINE-1 and Alu.* We used general linear regression models to estimate associations between LINE-1 and Alu DNA methylation and As exposure. For LINE-1, higher levels of As exposure were associated with increased methylation. In umbilical cord leukocytes (Figure 1A), LINE-1 methylation increased in the medium tertile and in the high tertile compared with the lowest tertile of drinking water As (Table 3). When As exposure was expressed using TUA/g, LINE-1 methylation in umbilical cord leukocytes (Figure 1A) also increased with As exposure although the relationship was significant only in the highest tertile of TUA/g (adjusted β = 1.36, SE = 0.42, *p* = 0.002) compared with the lowest tertile of TUA/g.

In maternal leukocytes (Figure 1B), LINE-1 methylation was not significantly associated with drinking-water As (Table 3). But LINE-1 methylation in maternal leukocytes was 1.08% higher among those in the highest tertile of TUA/g (adjusted β = 1.08, SE = 0.51, *p* = 0.04) compared with the lowest tertile.

No significant associations were observed between Alu methylation in umbilical cord leukocytes and drinking-water As (Table 3). Nor was there any association between umbilical cord Alu methylation and TUA/g. Nor were any significant associations observed between Alu methylation in maternal leukocytes and drinking-water As or when exposure was expressed as TUA/g.

p16 and p53. Associations between maternal As exposure and methylation of CpG sites within the promoter regions of *p16* and *p53* in umbilical cord leukocytes are presented in Table 4. When As exposure was categorized by drinking-water As, methylation at positions 2, 5, 6, and 7 within the promoter region of *p16* increased in the highest versus lowest tertile of As exposure. For example, methylation at position 2 increased 0.52% in association with the highest versus lowest tertile of drinking water after adjustment for maternal BMI and prematurity (β = 0.52, SE = 0.26, *p* = 0.05). Methylation at position 2 was also increased among those in the highest versus lowest tertile of creatinine-adjusted urinary As (β = 0.54, SE = 0.30, *p* = 0.07).

**Table 4:** Estimates and 95% confidence intervals (CIs) from general linear regression models that compared DNA methylation (%mC) in umbilical cord leukocytes according to tertiles of drinking-water As and TUA/g.

Drinking-water As (%mC)				TUA/g (%mC)			
Site	As tertile	(95% CI)		p-Value	(95% CI)		p-Value
p16												
pos1		High		–0.06	(–0.64, 0.53)		0.85		0.07	(–0.58, 0.73)		0.83
		Med		0.36	(–0.36, 1.08)		0.33		0.01	(–0.62, 0.65)		0.96
		Low		Ref					Ref			
pos2		High		0.52	(0.004, 1.04)		0.05		0.54	(–0.05, 1.12)		0.07
		Med		0.78	(0.15, 1.42)		0.02		0.16	(–0.42, 0.73)		0.59
		Low		Ref					Ref			
pos3		High		0.02	(–0.29, 0.34)		0.88		–0.01	(–0.36, 0.34)		0.98
		Med		0.11	(–0.28, 0.50)		0.58		–0.20	(–0.54, 0.14)		0.25
		Low		Ref					Ref			
pos4		High		0.31	(–0.08, 0.70)		0.12		0.21	(–0.23, 0.65)		0.35
		Med		0.33	(–0.15, 0.81)		0.17		–0.06	(–0.49, 0.37)		0.79
		Low		Ref					Ref			
pos5		High		0.50	(0.14, 0.86)		0.01		0.17	(–0.24, 0.58)		0.41
		Med		0.17	(–0.27, 0.61)		0.45		–0.15	(–0.54, 0.25)		0.46
		Low		Ref					Ref			
pos6		High		0.37	(0.68, 0.57)		0.02		0.15	(–0.20, 0.50)		0.40
		Med		0.18	(–0.20, 0.57)		0.34		0.16	(–0.19, 0.50)		0.37
		Low		Ref					Ref			
pos7		High		0.52	(0.07, 0.96)		0.02		0.33	(–0.18, 0.83)		0.20
		Med		0.39	(–0.16, 0.94)		0.16		–0.07	(–0.56, 0.42)		0.77
		Low		Ref					Ref			
p53												
pos1		High		0.22	(–0.18, 0.63)		0.27		0.30	(–0.18, 0.74)		0.19
		Med		–0.18	(–0.65, 0.30)		0.46		0.20	(–0.23, 0.64)		0.36
		Low		Ref					Ref			
pos2		High		–0.93	(–2.15, 0.30)		0.14		–0.27	(–1.64, 1.10)		0.70
		Med		–1.17	(–2.62, 0.28)		0.11		–0.35	(–1.69, 0.99)		0.61
		Low		Ref					Ref			
pos3		High		0.04	(–0.69, 0.48)		0.81		0.21	(–0.43, 0.85)		0.51
		Med		0.00	(–0.92, 0.46)		1.00		0.27	(–0.36, 0.89)		0.40
		Low		Ref					Ref			
pos4		High		–0.10	(–0.78, 0.59)		0.78		–0.21	(–0.97, 0.54)		0.57
		Med		0.00	(–0.82, 0.81)		0.99		–0.12	(–0.86, 0.62)		0.75
		Low		Ref					Ref			
Abbreviations: Med, medium; pos, position; Ref, reference. Models adjusted for maternal BMI (continuous) and prematurity (< 37 or ≥ 37 weeks gestation). Low tertile (≤ 1 μg/L); medium tertile (1–1.9 μg/L); high tertile (> 1.9 μg/L).												

The association between As exposure and methylation in *p16* and *p53* in maternal blood are presented in Table 5. In maternal blood, methylation at CpG positions 1, 2, 3, and 6 in *p16* was associated with creatinine-adjusted urinary As, although except for position 3, associations were stronger for the medium versus highest tertile of TUA/g. Methylation of the CpG site at position 3 was also increased for highest versus lowest tertile of drinking-water As (β = 0.28, SE= 0.14, *p* = 0.05 for the highest tertile of drinking-water As, and β = 0.34, SE = 0.15, *p* = 0.03 for the highest tertile of TUA/g.).

**Table 5:** Estimates and 95% confidence intervals (CIs) from general linear regression models that compared DNA methylation (%mC) in maternal leukocytes according to tertiles of drinking-water As and TUA/g.

As tertile	Drinking-water As (%mC)				TUA/g (%mC)			
	Site	β (95% CI)		p-Value	β (95% CI)		p-Value
p16												
pos1		High		–0.01	(–0.70, 0.68)		0.97		0.46	(–0.27, 1.20)		0.21
		Med		–0.28	(–1.13, 0.58)		0.53		1.00	(0.28, 1.73)		0.01
		Low		Ref					Ref			
pos2		High		0.27	(–0.48, 1.02)		0.47		0.73	(–0.07, 1.53)		0.07
		Med		–0.08	(–1.01, 0.85)		0.86		1.13	(0.35, 1.92)		0.01
		Low		Ref					Ref			
pos3		High		0.28	(0, 0.57)		0.05		0.34	(0.03, 0.65)		0.03
		Med		0.07	(–0.28, 0.42)		0.69		0.27	(–0.04, 0.57)		0.08
		Low		Ref					Ref			
pos4		High		0.13	(–0.32, 0.59)		0.56		0.30	(–0.16, 0.83)		0.24
		Med		–0.10	(–0.67, 0.46)		0.72		0.38	(–0.08, 0.89)		0.14
		Low		Ref					Ref			
pos5		High		0.06	(–0.26, 0.38)		0.70		0.10	(–0.25, 0.45)		0.58
		Med		–0.22	(–0.62, 0.17)		0.27		0.29	(–0.05, 0.64)		0.10
		Low		Ref					Ref			
pos6		High		0.19	(–0.10, 0.49)		0.20		0.29	(–0.03, 0.61)		0.07
		Med		–0.01	(–0.38, 0.36)		0.96		0.38	(0.06, 0.69)		0.02
		Low		Ref					Ref			
pos7		High		0.53	(–0.40, 1.47)		0.26		0.48	(–0.56, 1.52)		0.36
		Med		–0.40	(–1.57, 0.77)		0.50		0.78	(–0.25, 1.80)		0.13
		Low		Ref					Ref			
p53												
pos1		High		–0.23	(–1.03, 0.57)		0.57		–0.21	(–1.11, 0.69)		0.64
		Med		–0.19	(–1.21, 0.84)		0.72		–0.61	(–1.50, 0.29)		0.18
		Low		Ref					Ref			
pos2		High		–0.29	(–1.52, 0.94)		0.64		–0.61	(–2.00, 0.77)		0.38
		Med		–0.15	(–1.72, 1.42)		0.85		–0.59	(–1.97, 0.79)		0.40
		Low		Ref					Ref			
pos3		High		0.08	(–0.38, 0.54)		0.73		–0.04	(–0.56, 0.47)		0.87
		Med		–0.20	(–0.79, 0.38)		0.49		–0.30	(–0.81, 0.22)		0.25
		Low		Ref					Ref			
pos4		High		0.48	(0.34)		0.16		0.31	(0.39)		0.43
		Med		–0.01	(0.44)		0.97		–0.24	(0.39)		0.54
		Low		Ref					Ref			
Abbreviations: Med, medium; pos, position; Ref, reference. Models adjusted for maternal BMI. Low tertile (≤ 0.35 μg/g); medium tertile (0.35 – 0.66 μg/g); high tertile (> 0.66 μg/g).												

No significant associations were observed between As exposure and methylation at any of the 4 CpG sites measured in *p53* in either umbilical cord or maternal leukocytes (Tables 4 and 5).

## Discussion

In our study population, As exposure measured before the 25th week of gestational age was associated with modest increases in CpG methylation in LINE-1 repeated elements measured in DNA extracted from leukocytes of healthy pregnant women and their newborns. Methylation at some of the CpG sites within the promoter region of *p16* also increased with As exposure, although the strongest associations were in the middle tertiles in all but one position, which could indicate a nonlinear dose–response relationship. This leads to speculation that moderate levels of arsenic exposure may increase methylation within the promoter region of *p16* in pregnant women and their infants although additional experimental and epidemiological studies are needed to validate these findings.

Although this is the first epidemiological study to observe an association between moderate As exposure and DNA methylation in newborns, previous experimental studies have shown that high levels of As can alter DNA methylation. Cell culture experiments in human lung, kidney, and keratinocytes show that As increases methylation within the promoter regions of the tumor suppressor gene *p53*, the noncanonical WNT signaling pathway and the death-associated protein kinase (*DAPK*) gene, but reduces global DNA methylation (Chen et al. 2004; Chen et al. 2007; Christensen et al. 2007; Huang et al. 2011; Jensen et al. 2008, 2009; Mass and Wang 1997; Xie et al. 2007; Zhao et al. 1997; Zhong and Mass 2001). In mice, chronic exposure to As induced global DNA hypomethylation, estrogen-receptor α (ERα) hypomethylation, and up-regulation of ERα expression (Chen et al. 2004).

Epidemiological studies in adults have also observed associations between As and DNA methylation. In a bladder cancer study conducted in New Hampshire (USA), tumor suppressor genes *RASSF1A* and *PRSS3* were hypermethylated in bladder cancer tumors collected from 18 individuals who had toenail As levels at or above the 95th percentile compared with 318 tumors from individuals with toenail arsenic levels below the 95th percentile (Marsit et al. 2006). In adults participating in a 12-week folic acid supplementation trial (*n* = 294) in Bangladesh, global DNA methylation in leukocytes increased with urinary arsenic levels among 104 folate replete adults (Pilsner et al. 2007). In a skin cancer study in West Bengal, India, 72 adults who were exposed to As and had dermal symptoms of chronic As toxicity had higher DNA methylation of the promoter regions of *p53* and *p16* than 24 unexposed individuals (Chanda et al. 2006). Additionally, DNA methylation in leukocytes appeared to have a nonlinear exposure–response relationship, such that global methylation increased when drinking water As concentrations were 250–500 μg/L but then decreased when As was > 500 μg/L (Majumdar et al. 2010). In Guizhou, China, a case–control study reported that methylation of the promoter region of *p16* was increased in 103 adult arsenicosis patients compared with 110 controls, and that *p16* hypermethylation increased with the severity of the arsenic-related skin lesion (Zhang et al. 2007). Furthermore, a small case–control study performed a genome-wide screen of DNA methylation patterns and identified 182 hypermethylated genes in peripheral lymphocyte DNA in 8 adults with arsenic-related skin lesions compared with 8 adults without arsenic-related skin lesions (Smeester et al. 2011).

Although it is not known whether the small differences in leukocyte methylation in LINE-1 or *p16* observed in this study are associated with any adverse health effects, transcriptional silencing through hypermethylation of tumor-suppressor gene promoters and chromosomal instability from aberrant methylation of repeated elements are consistent with the epigenetic events observed in carcinogenesis (Feinberg and Tycko 2004; Ogino et al. 2008). Furthermore, inactivation of *p16*, by methylation, which is frequently detected in tumor tissues, has led to speculation that loss of cell cycle control resulting from methylation of *p16* may be an early biomarker of carcinogenesis (Attri et al. 2005; Gazzeri et al. 1998).

An important consideration for this study is that blood contains a mixture of different white blood cell types and umbilical cord blood can contain stem cells. Arsenic affects erythropoiesis (Saulle et al. 2006) and may affect white blood cell populations (Parmar and Tallman 2003). Because we analyzed DNA from whole blood we are capturing a mixture of leukocytes, and the different responses we observed between mothers and their infants may be attributable to differing white blood cell subpopulations or stem cells. Other limitations of this study are its small sample size; the skewed distribution of As exposures, which makes it difficult to discern substantial differences between the low and medium tertiles of As exposure; and As exposure measurements at only one point in time during early pregnancy. Also, we could adjust for the dilution of urine using only creatinine because we did not measure the specific gravity of urine. This is problematic because creatinine, arsenic metabolism, and DNA methylation are all mediated by one-carbon metabolism (Pilsner et al. 2009). Subsequently, adjusting total urinary arsenic for creatinine, instead of specific gravity, could be a source of bias in this study.

However, previous research has shown that arsenic can alter DNA methylation, and it is biologically plausible that early pregnancy may be a critical window for exposure to epigenetic toxicants because methylation marks are being established in the fetus. Additionally, we used both environmental and personal measurements to characterize As exposure. All methylation assays were run in a single batch to reduce analytical variability, and technicians were blinded to exposure. Finally, pyrosequencing is a sensitive method that quantifies very small changes in DNA methylation at specific loci and sequences in the genome. Other commonly used DNA methylation assays might not have been able to detect these small changes.

In conclusion, this study showed an association between moderate drinking-water As exposure and increased DNA methylation in LINE-1, and to a lesser extent increased methylation within the promoter region of *p16,* in leukocytes in healthy pregnant women and their infants. Additional research on low-level arsenic exposure is needed to confirm these findings and determine whether these small changes in DNA methylation are associated with any adverse health effects. It is also critically important that efforts continue to reduce As exposure from contaminated drinking water in Bangladesh.

## Supplemental Material

(422 KB) PDF
