# Supplementary material for: Prenatal Arsenic Exposure and DNA Methylation in Maternal and Umbilical Cord Blood Leukocytes
Source: Environ Health Perspect. 2012 Mar 30;120(7):1061–6. doi: 10.1289/ehp.1104173 (PMC3404653; doi:10.1289/ehp.1104173)
Supplement: (422 KB) PDF [file ehp.1104173.s001.pdf]

Supplementary Material Table 1: Location of gene promoters and regions that were amplified and the CpG dinucleotide positions within the regions where DNA methylation was quantified.

Gene	Chromosome	Promoter		Amplicon		CpGs
		Start	***End***	***Start***	***End***	
**P16**	9	21964701	21965538	21965321	21965395	21965350 (position 1)<br>21965355 (position 2)<br>21965357 (position 3)<br>21965361 (position 4)<br>21965365 (position 5)<br>21965368 (position 6)<br>21965374 (position 7)
**P53**	17	7531143	7531743	7531409	7531628	7531486 (position 1)<br>7531473 (position 2)<br>7531469 (position 3)<br>7531458 (position 4)

Supplementary Material Table 2: Primers used for DNA methylation analysis

ID	Forward Primer (5' to 3')	Reverse Primer (5' to 3')	Sequencing Primer (5' to 3')	Sequence analyzed[a]
Global methylation analysis				
Alu	Biotin-TTTTTATTAAAAATATAAAAATT	CCCAAACTAAAATACAATAA	AATAACTAAAATTACAAAC	G/AC/TG/AC/TG/ACCACCA
LINE-1	TTTTGAGTTAGGTGTGGGATATA	Biotin-AAAATCAAAAAATTCCCTTTC	AGTTAGGTGTGGGATATAGT	TTC/TGTGGTGC/TGTC/TG
Gene-specific methylation analysis				
p16	AGGGGTTGGTTGGTTATTAG	Biotin - CTACCTACTCTCCCCCTCTC	GGTTGGTTATTAGAGGGT	GGGGC/TGGATC/TGC/TGTGC/TGTTC/TGGC/TGGTTGC/TG
p53	Biotin - TTAGGAGTTTATTTAATTTAGGGAAG	TATCCAACTTTATACCAAAAACCTC	TCCAAAAAACAAATAACTACTAAACTC	CG/AAAAACACTTTACG/ATTCG/AAACTAAAAACG/ATACTTT

[a]Nucleotides at which DNA methylation was measured are underlined

Supplementary Material Table 3. Estimates and 95% confidence intervals from general linear regression models that compared DNA methylation (%mC) in umbilical cord blood and maternal blood according to tertiles of total urinary arsenic (μg/L). Models are adjusted for maternal BMI and creatinine. Additionally, cord blood models are adjusted for fetal age.

		Cord Blood			Maternal Blood		
		β	95% CI	p-value	β	95% CI	p-value
LINE-1	High	0.95	-0.09, 1.99	0.07	0.06	-1.17, 1.30	0.92
	Med	0.32	-0.53, 1.17	0.46	0.05	-0.99, 1.10	0.92
	Low	Ref.	-	-	Ref.	-	-
Alu	High	0.14	-0.30, 0.58	0.52	-0.12	-0.54, 0.31	0.59
	Med	0.02	-0.34, 0.38	0.92	0.24	-0.12, 0.60	0.19
	Low	Ref.	-	-	Ref.	-	-
P16 pos 1	High	0.42	-0.37, 1.21	0.29	0.27	-0.64, 1.18	0.55
	Med	0.04	-0.62, 0.69	0.91	-0.01	-0.79, 0.76	0.97
	Low	Ref.	-	-	Ref.	-	-
P16 pos 2	High	0.64	-0.06, 1.35	0.07	0.47	-0.52, 1.46	0.5
	Med	-0.03	-0.62, 0.55	0.91	0.17	-0.67, 1.02	0.74
	Low	Ref.	-	-	Ref.	-	-
P16 pos 3	High	0.22	-0.21, 0.64	0.32	0.27	-0.10, 0.64	0.15
	Med	-0.02	-0.38, 0.33	0.89	-0.02	-0.33, 0.29	0.9
	Low	Ref.	-	-	Ref.	-	-
P16 pos 4	High	0.4	0.13, 0.92	0.14	0.38	-0.22, 0.98	0.98
	Med	-0.09	0.52, 0.35	0.7	0.35	-0.15, 0.86	0.86
	Low	Ref.	-	-	Ref.	-	-
P16 pos 5	High	0.22	-0.27, 0.71	0.38	0.09	-0.33, 0.52	0.66
	Med	-0.23	-0.64, 0.17	0.26	0.13	-0.24, 0.49	0.49
	Low	Ref.	-	-	Ref.	-	-
P16 pos 6	High	0.16	-0.26, 0.58	0.44	0.22	-0.17, 0.62	0.26
	Med	-0.23	-0.58, 0.12	0.2	0.07	-0.26, 0.40	0.67
	Low	Ref.	-	-	Ref.	-	-
P16 pos 7	High	0.37	-0.23, 0.97	0.22	0.55	-0.70, 1,80	0.39
	Med	-0.31	-0.81, 0.19	0.22	0.41	-0.66, 1.48	0.45
	Low	Ref.	-	-	Ref.	-	-
P53 pos 1	High	0.29	-0.24, 0.82	0.28	-0.05	-1.09, 1.00	0.93
	Med	-0.10	-0.55, 0.35	0.66	-0.58	1.52, 0.36	0.22
	Low	Ref.	-	-	Ref.	-	-
P53 pos 2	High	-0.32	-1.96, 1.33	0.7	-0.06	-1.67, 1.56	0.94
	Med	0.39	-1.78, 1.00	0.58	-0.50	-.95, 0.95	0.5
	Low	Ref.	-	-	Ref.	-	-
P53 pos 3	High	0.19	-0.57, 0.96	0.62	-0.09	-0.70, 0.52	0.77
	Med	0.17	-0.48, 0.82	0.6	-0.11	-0.66, 0.43	0.68
	Low	Ref.	-	-	Ref.	-	-
p53 pos 4	High	-0.27	-1.16, 0.63	0.55	0.27	-0.64, 1.17	0.56
	Med	-0.42	-1.18, 0.33	0.27	0.38	-0.43, 1.19	0.36
	Low	Ref.	-	-	Ref.	-	-
